# Characterization of Holographic Gratings in PVA/AA Using Coherent Nanosecond Laser Exposure

**DOI:** 10.3390/polym17212873

**Published:** 2025-10-28

**Authors:** Emilio J. Mena, Andrés P. Bernabeu, Guillem Nájar, Sergi Gallego, Andrés Márquez, Augusto Beléndez

**Affiliations:** 1 Instituto Universitario de Física Aplicada a las Ciencias y las Tecnologías, Universidad de Alicante, E-03080 Alicante, Spain; guillem.najar@ua.es (G.N.); sergi.gallego@ua.es (S.G.); andres.marquez@ua.es (A.M.); a.belendez@ua.es (A.B.); 2 Departamento de Física, Ingeniería de Sistemas y Teoría de la Señal, Universidad de Alicante, E-03080 Alicante, Spain; andres.perezber@ua.es

**Keywords:** holography, PVA/AA, pulsed laser, photopolymer, yellowish eosin

## Abstract

This work investigates the performance of a polyvinyl alcohol/acrylamide-based photopolymer (PVA/AA) under pulsed and continuous-wave (CW) laser exposure for holographic recording. Using a versatile setup that allows direct comparison between both regimes, diffraction efficiency (DE), angular responses, and material parameters were analyzed. Comparable maximum DE was achieved with both CW and pulsed laser exposure; however, the pulsed regime produces stronger attenuation along the grating depth, which emphasizes the need to lower the dye concentration. Furthermore, the temporal evolution of DE reveals a smaller influence of oxygen inhibition under CW exposure. Finally, second-order angular reconstructions confirm that pulsed gratings exhibit a better sinusoidal modulation, especially at low frequency rates. These results enhance the understanding of how pulsed exposure affects holographic recording and open pathways for optimizing photopolymer systems in advanced technologies.

## 1. Introduction

The development of new recording materials has been a key factor in the expansion of holographic applications such as holographic data storage, holographic displays, and holographic sensors. In particular, photopolymers have become an efficient alternative to other traditional methods based on silver halides due to their multiple advantages, including ease of handling, low cost, chemical stability, and high performance [1,2,3]. However, these materials also present certain drawbacks, such as oxygen inhibition. During the initial recording stage, energy is delivered to the material, but polymerization does not occur immediately due to the presence of dissolved oxygen. The photoinitiated radicals generated in the early stages must first react with oxygen molecules before chain propagation can begin. As a result, an inhibition time arises in which photopolymerization does not proceed, increasing the energy required to initiate the process. Several strategies have been proposed to minimize this effect, including the incorporation of additional dyes, avoiding the presence of oxygen during preparation, or pre-illuminating the sample before recording [2,4,5,6,7].

Among these materials, the polyvinyl alcohol/acrylamide (PVA/AA) photopolymer stands out due to its easy preparation, high efficiency, good angular selectivity, and simple bleaching process [8,9,10,11]. Numerous studies have shown that this material can store high-efficiency and temporally stable holograms when exposed to continuous-wave (CW) light sources. Also when nanoparticles are incorporated into the composition, improved performance has been reported [12,13]. However, its behavior under pulsed laser conditions remains a less explored area. Nevertheless, it has been demonstrated that, under pulsed recording conditions using the copying method, the dye concentration must be reduced compared to CW illumination [14]. Furthermore, the addition of MBA as a crosslinker in PVA/AA photopolymer significantly improves the diffraction efficiency during pulsed-laser recording and reduces the energy threshold required to initiate polymerization [15]. Pulsed light has also been applied to study phase-shift dynamics in PVA/AA holograms [16] and to enable the recording of moving objects and ultrafast processes [17].

To complement these experimental findings, theoretical models have been developed to describe polymerization dynamics in photopolymers. Early approaches assumed a local response, where photopolymerization at a point depended only on the local light intensity. In 2000, a nonlocal response model associated with chain growth was proposed [18], in which chain formation probability is expressed as a convolution with a Gaussian function. Based on this concept, a nonlocal polymerization-driven diffusion (NPDD) model was developed and experimentally validated [19,20,21]. This model revealed that, after short exposures, grating amplification persists even after illumination ceases because of monomer diffusion and continued chain growth. These insights highlight the potential of pulsed lasers as a tool to refine such models by probing the processes occurring during the dark intervals between pulses, thereby allowing for a more detailed control of the propagation kinetics of photopolymeric materials.

In this context, several studies have already been carried out. For instance, acrylamide has been found to exhibit faster propagation kinetics than methacrylamide in aqueous solutions, with its propagation coefficient, $k_{c}$, increasing as the temperature decreases [22]. Moreover, a pulsed holographic setup enabled the observation of intermediate gratings, characterized by microsecond lifetimes, that preceded the formation of permanent and stable gratings in PVA/AA photopolymers containing erythrosine and ethanol [23]. In addition, other studies have explored the use of nanosecond pulsed lasers in different materials. For example, the holographic performance of PQ/PMMA was investigated using the dark diffusion enhancement process (DDEP), revealing that the grating strength increases after exposure due to molecular diffusion of PQ from unexposed to exposed regions. This provides deeper insight into the fast photopolymerization dynamics occurring in such systems [24].

Therefore, pulsed exposure introduces significant differences compared to CW illumination, with pulse repetition frequency acting as an additional control parameter. Understanding how the PVA/AA system responds under these conditions is essential, as this knowledge offers a deeper understanding of the behavior of this material and, more broadly, of photopolymeric systems in general. In this work we present an experimental setup specifically designed to study these effects in a simple and controlled manner, maintaining identical recording conditions for both CW and pulsed regimes. The main aim of this study is to evaluate the performance of this photopolymer with pulsed holographic recording and to compare the results with those obtained under continuous exposure by analyzing the diffraction efficiency and hologram quality. To this end, second-order Bragg diffraction will be examined since a weaker second-order diffraction is indicative of a more purely sinusoidal pattern [25,26,27].

The structure of the paper is as follows. Section 2 describes the experimental setup used to record and reconstruct the gratings under both CW and pulsed exposure, as well as the different component concentrations employed in the preparation of the photopolymer. Section 3 presents the results together with a concise discussion. This section is divided into three parts: first, the effect of the dye concentration, particularly under pulsed exposure, is analyzed. Next, a comparison between CW and pulsed exposure gratings at different repetition rates is presented. A similar total exposure energy was maintained to study the differences observed both during recording and in the angular reconstructions after bleaching. Finally, second-order angular reconstructions are examined in order to investigate grating nonuniformities. The paper concludes in Section 4 with a summary of the main findings.

## 2. Experimental Setup and Materials

**Figure 1:**
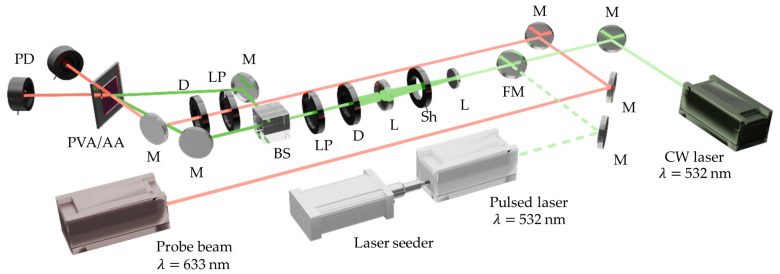
Experimental setup. PD: Photodetector. D: Diaphragm. LP: Linear polarizer. BS: Beamsplitter. L: Lens. Sh: Shutter. M: Mirror. FM: Flip mirror.

One of the main advantages of the experimental setup used in this work (shown in Figure 1) is its versatility for comparing continuous-wave and pulsed exposure conditions. The system consists of a coherent nanosecond pulsed laser whose emission is divided by a beamsplitter into two beams, which are subsequently recombined in the plane of the sample to form the interference pattern required for holographic recording.

The design of the setup enables straightforward integration of a CW laser aligned along the same optical path via a flip mirror mount. This configuration allows for easy selection between pulsed or CW beams without modifying the system alignment, while all other optical elements remain fixed. Consequently, holographic recordings in both regimes are performed under identical geometric conditions, ensuring a direct and reliable comparison of the results.

Samples are exposed using a collimated beam from a frequency-doubled Nd:YAG Q-switched laser (Quantel Q-smart-450, Lumibird, Lannion, France) with a pulse duration of 5 ns and a maximum repetition rate of 10 Hz. This laser incorporates an NP Photonics Rock Fiber Laser Seeder, responsible for the long coherence length (>30 cm) of the pulsed wavefronts. For continuous-wave recording, a diode-pumped solid-state (DPSS) laser (Oxxius L1C-MPA-532, Lumibird, Lannion, France) is used. In both regimes, the reference and object beams are symmetrically incident on the material at an angle of 23.5° relative to the normal, resulting in spatial frequencies of approximately 1500 lines/mm for the 532 nm wavelength. For hologram reconstruction, a supercontinuum white-light laser (NKT Photonics SuperK EVO), combined with a wavelength selector (NKT Photonics SuperK VARIA) tuned to 633 nm with a 1 nm spectral bandwidth, is incident at the Bragg angle (28.3°). This is because the dye is not sensitive to this part of the electromagnetic spectrum.

The pulsed laser output power is measured with a Newport 919P-003-10 High Sensitivity Thermopile Sensor (Julian Camarillo, Madrid, Spain), whereas the CW laser, as well as the transmitted and diffracted beams, are monitored using a Newport 918D-SL-OD3R Silicon Photodetector (Julian Camarillo, Madrid, Spain). All detectors are connected to a Newport Optical Power Meter (Model 2936-C, Julian Camarillo, Madrid, Spain). It is important to note that all beams employed in the experiments are TE-polarized to ensure optimal fringe contrast during recording and to maximize diffraction efficiency during reconstruction.

Regarding the photopolymer composition, it is made up of polyvinyl alcohol 18-88 (PVA) as a binder, acrylamide (AA) as a monomer, N,N′methylene-bis-acrylamide (MBA) as a crosslinker, triethanolamine (TEA) as a photoinitiator, and yellowish eosin (YE) as a dye. All components were supplied by Sigma-Aldrich. To study the influence of the dye, three different YE concentrations were prepared, summarized in Table 1.

Once prepared, 1 mL of the photopolymer solution was deposited onto glass plates covering an area of 4 × 4 cm^2^, resulting in films with a thickness of approximately 92 µm. The films were then left to dry for 24 to 48 h until they solidified into a stable layer and were subsequently used for recording. After holographic grating formation, the samples were bleached under ambient light for 24 to 48 h to ensure complete consumption of residual dye.

## 3. Results and Discussion

### 3.1. Dye Optimization

To investigate the effect of the dye concentration under CW and pulsed recording conditions, gratings were recorded using the three different photopolymer compositions listed in Table 1. For CW exposure, a power density of 8.16 mW/cm^2^ was applied for 7 s for Type 1 and Type 2, and 8 s for Type 3, corresponding to total exposures of 57.12 mJ/cm^2^ and 65.28 mJ/cm^2^, respectively. For pulsed exposure, 92 pulses were applied at a repetition rate of 10 Hz, with a fluence of 0.70 mJ/cm^2^ per pulse, resulting in a total exposure of 64.40 mJ/cm^2^. In all cases, exposure was deliberately stopped after reaching the maximum DE, slightly overmodulating the grating since the subsequent bleaching process leads to a partial recovery of efficiency from the registration process. It should be noted that both the CW and the pulsed laser recordings were performed with very similar power density values to facilitate the comparison of the results.

**Figure 2:**
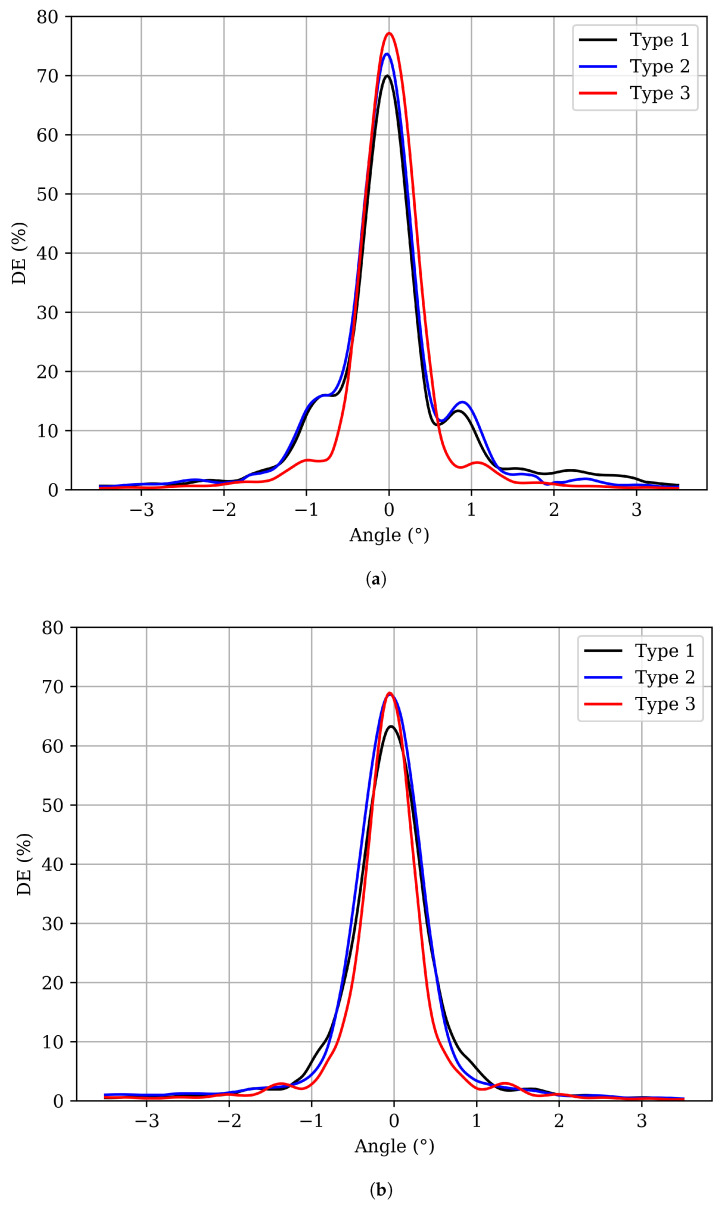
Angular reconstruction of photopolymer samples with different YE concentrations after bleaching, exposed under (**a**) CW laser: power density of 8.16 mW/cm^2^, exposure time of 7 s for Type 1 and Type 2, and 8 s for Type 3, corresponding to a total exposure of 57.12 mJ/cm^2^ and 65.28 mJ/cm^2^, respectively; (**b**) pulsed laser: pulse fluence of 0.70 mJ/cm^2^, 92 pulses at 10 Hz, and total exposure of 64.40 mJ/cm^2^.

Angular responses of the cured samples are shown in Figure 2. In all the cases studied, the maximum diffraction efficiency reaches approximately 70%, mainly limited by Fresnel reflection losses and by the relatively high spatial frequencies for PVA/AA photopolymer [28].

In the case of CW laser exposure, a high YE concentration results in excessive DE between the central maximum and the secondary lobes, preventing the curve from reaching zero. When the dye concentration is reduced, this inter-lobe efficiency decreases significantly, leading to a cleaner angular response. In contrast, gratings recorded under pulsed exposure exhibit a strong reduction, or even disappearance, of secondary lobes, indicating a higher attenuation along the grating depth compared to the CW exposure [29]. Additionally, we observe that the width of the central maximum is clearly smaller in the case of CW exposure than for pulsed illumination.

This behavior can be attributed to two main factors. First, under pulsed conditions, the dye concentration must be reduced relative to CW exposure to avoid excessive absorption near the surface and, therefore, to achieve uniform grating formation throughout the film. High dye concentrations in pulsed regimes lead to strong surface polymerization and limited depth penetration [14]. Second, although previous studies have shown that monomer diffusion in depth during and after illumination is minimal under CW exposure [30], the situation appears to differ under pulsed illumination. The presence of dark intervals between pulses seems to allow monomer distribution from deeper layers towards the surface. This upward migration could explain the experimental observations, resulting in a nonuniform concentration profile with greater accumulation near the surface. Consequently, polymerization becomes more localized in the upper regions of the film, increasing attenuation and thus reducing the optical thickness. Together, these effects highlight the critical interplay between the dye concentration and monomer dynamics in achieving uniform volume grating formation under pulsed laser conditions.

Based on Rigorous Coupled-Wave (RCW) theory [29,31], the effective refractive index modulation, Δn, and the attenuation, α, were determined for samples exposed to pulsed light. In our photopolymer layers, scattering-induced attenuation is negligible; therefore, α primarily reflects absorption effects that arise from the exponential attenuation of light within the material, as described by Beer’s law. This attenuation imposes a practical limit on the physical thickness of the layer, defining the optical thickness, which serves as one of the key parameters for characterizing volume diffraction gratings. As summarized in Table 2, Δn remains nearly constant across the three cases studied, consistent with the similar exposure conditions. In contrast, attenuation of the grating along the depth of the material exhibits a significant variation as the dye concentration decreases, leading to an increase in optical thickness. This increase, estimated using Kogelnik’s theory, results in the reappearance of secondary lobes in the angular response, separated from the main lobe, as shown in Figure 2b. It should be noted that the values of Δn, α, and $d_{\mathrm{optic}}$ were obtained by iteratively fitting the experimental angular reconstruction curves to the theoretical predictions until satisfactory agreement was achieved.

### 3.2. Direct Comparison Between Continuous-Wave and Pulsed Exposure

The effect of varying the dark interval between pulses was investigated using the Type 3 composition. For this purpose, the pulse fluence was kept constant, while the repetition rate was modified, and the results were compared with those obtained under continuous-wave exposure. To ensure a fair comparison, all holographic recordings were performed with total accumulated energies as similar as possible in both regimes. In the CW case, a power density of 8.16 mW/cm^2^ was applied for 8 s, resulting in a total exposure of 65.28 mJ/cm^2^. For pulsed exposure, a pulse fluence of 0.854 mJ/cm^2^ and a total of 76 pulses were used, leading to a total exposure of 64.90 mJ/cm^2^. It should be noted that, since the repetition rates differ, the same number of pulses corresponds to different total exposure times.

**Figure 3:**
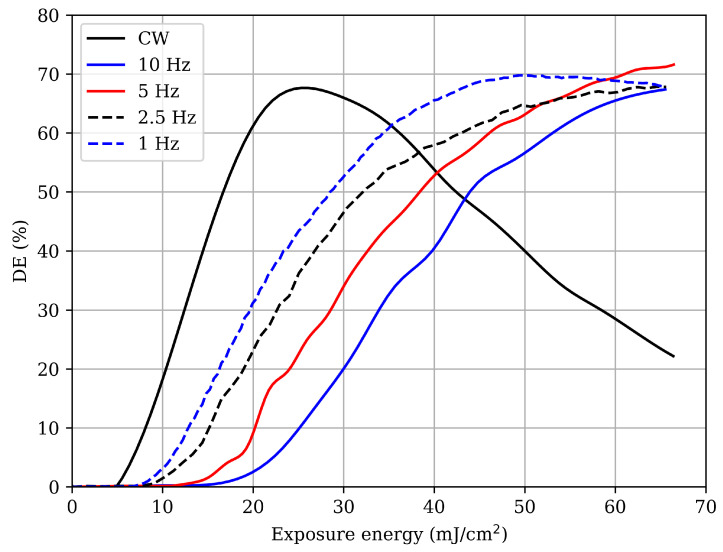
Diffraction efficiency during recording as a function of the accumulated energy per area for photopolymer Type 3. CW: power density of 8.16 mW/cm^2^, exposure time of 8 s, and total exposure of 65.28 mJ/cm^2^; pulsed: pulse fluence of 0.854 mJ/cm^2^, 76 pulses at different repetition rates, and total exposure of 64.90 mJ/cm^2^.

Figure 3 shows the evolution of DE as a function of the accumulated energy per unit area for both CW and pulsed exposures. At the beginning of recording, an inhibition period is observed during which polymerization does not yet occur because the available energy is primarily consumed in oxygen depletion. Once the oxygen is fully consumed, polymerization starts, and DE increases. The earlier rise of the CW curve compared to the pulsed ones indicates that less accumulated energy is required to overcome the inhibition barrier under CW illumination. In contrast, pulsed exposure produces a more pronounced inhibition effect, and the repetition rate becomes a key factor in the material response. At higher pulse frequencies, more accumulated energy is required to initiate polymerization, whereas at lower frequencies the inhibition threshold decreases, allowing an earlier onset of grating formation. While CW exposure produces a faster increase in DE followed by overmodulation, pulsed exposure results in a slower and smoother evolution. At the lowest repetition rates, the DE curve even exhibits a plateau near its maximum value (see the case for 1 Hz in Figure 3), where efficiency remains nearly constant for a certain interval, suggesting limited overmodulation capability.

**Figure 4:**
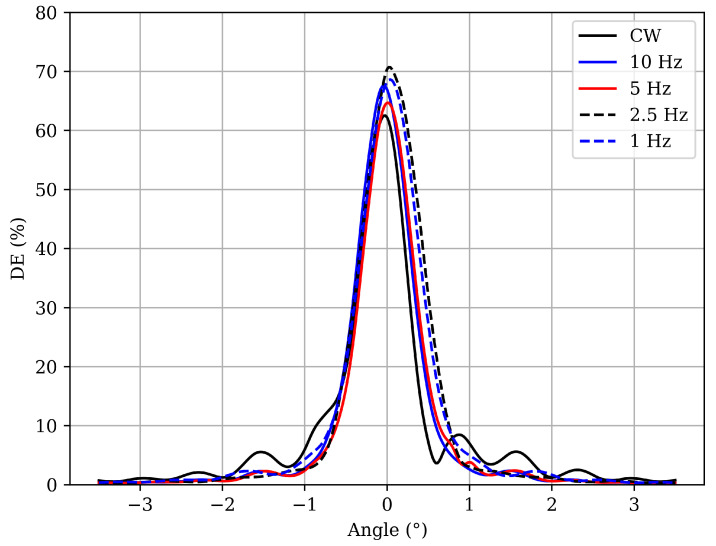
Angular reconstruction of photopolymer Type 3 exposed with different repetition rates after bleaching. CW: power density of 8.16 mW/cm^2^, exposure time of 8 s, and total exposure of 65.28 mJ/cm^2^; pulsed: pulse fluence of 0.854 mJ/cm^2^, 76 pulses at different repetition rates, and total exposure of 64.90 mJ/cm^2^.

After bleaching, the angular reconstructions of the gratings were obtained (Figure 4). All samples recorded under pulsed exposure maintain diffraction efficiencies close to 70%. Notably, the sample recorded under CW illumination exhibits a significant increase in DE after curing, resulting in a grating that appears less overmodulated than in its initial state. All samples were exposed on the same day to ensure identical laboratory conditions. Furthermore, the CW-exposed grating in Figure 3 and the Type 3 grating shown in Figure 2 were exposed with identical parameters but at different times, demonstrating the high repeatability of the experimental procedure, despite minor variations in ambient temperature, humidity, composition, or film thickness.

In Table 3 the fitting parameters obtained from the angular reconstruction curves are presented. Although photopolymerization under CW exposure proceeds faster, leading to overmodulation of the grating, the effective refractive index modulation remains nearly constant in both regimes after bleaching. This suggests that polymerization is largely governed by the total accumulated energy rather than by the temporal structure of the exposure, i.e., the way energy is delivered to the sample. Meanwhile, the attenuation parameter increases progressively as the pulse repetition rate decreases, with CW exposure exhibiting the lowest value. Consequently, the optical thickness shows a slight reduction with the decrease in repetition rate while remaining highest under CW light. These results demonstrate that, although Δn is largely preserved, frequency-dependent attenuation in pulsed exposure limits the effective depth of the grating. Overall, CW exposure promotes deeper polymerization, whereas low-frequency pulsed exposure results in thinner effective gratings, highlighting the crucial role of temporal exposure dynamics in determining the optical properties of photopolymer holographic gratings.

### 3.3. Second Diffraction Order

**Figure 5:**
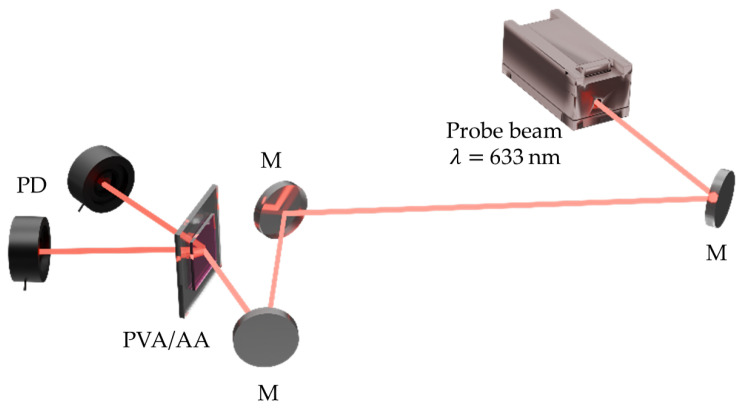
Experimental setup to measure second diffraction order. PD: photodetector. M: mirror.

The angular response of the second diffraction order was measured using the experimental setup shown in Figure 5, with a He–Ne laser (REO 30995, 633 nm) operating at a power of approximately 10 mW. In this configuration, the beam is incident on the holographic grating in the second harmonic of the Bragg condition, which theoretically corresponds to an incidence angle of about 71° relative to the normal.

**Figure 6:**
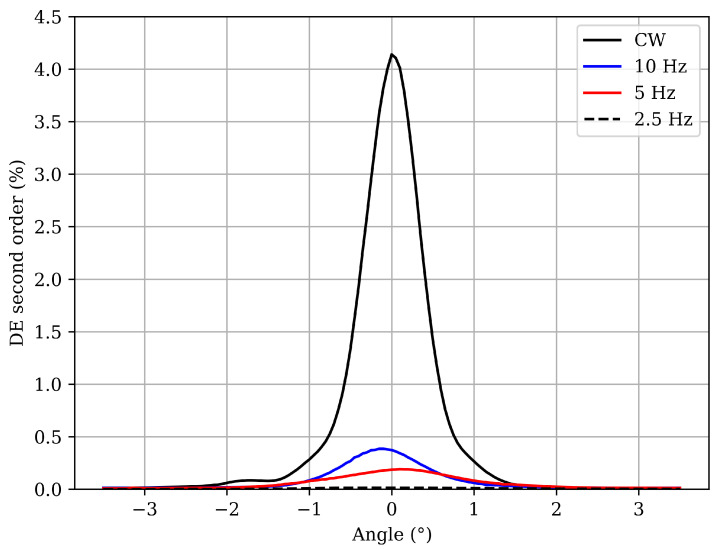
Angular reconstruction of the second diffraction order for the gratings presented in Figure 3 and Figure 4 and Table 3.

Figure 6 shows the angular reconstructions around the second-order Bragg angle for the gratings presented in Figure 3 and Figure 4 and Table 3. A clear trend is observed: as the pulse repetition rate decreases, the diffraction efficiency of the second harmonic is also reduced. The results reveal differences of up to two orders of magnitude between the grating recorded under CW illumination and that obtained with pulsed exposure at 2.5 Hz. Previous studies [26] have demonstrated both theoretically and experimentally that certain photopolymers exposed to high irradiance conditions exhibit weaker first-order and stronger higher-order diffractions. However, Figure 6 shows that pulsed laser exposure mitigates these nonlinear effects and that reducing the repetition rate further decreases the second-order Bragg diffraction efficiency.

In pulsed exposure, the presence of dark intervals between successive pulses plays a crucial role in the recording process, as monomer diffusion can occur during these periods. Since diffusion dominates over polymerization during the dark intervals, the refractive index profiles become more linear, leading the gratings to approach an ideal sinusoidal modulation. This behavior is consistent with the findings of [27], which demonstrated that gratings exhibiting a more sinusoidal pattern correspond to lower second-order diffraction efficiencies, a result attributed to the dominance of diffusion. In that work, a parameter $R_{D}$ was introduced to quantify the relative contribution of diffusion and polymerization processes. When $R_{D}>1$, diffusion dominates, whereas for $R_{D}<1$, polymerization prevails. The authors further showed that the more sinusoidal the grating profile, the higher the value of $R_{D}$.

## 4. Conclusions

Both continuous-wave (CW) and pulsed exposure provide similar maximum diffraction efficiency (DE) values of approximately 70% (without Fresnel correction) and comparable refractive index modulation, Δn. However, the results clearly demonstrate that the behavior of PVA/AA photopolymers under pulsed exposure differs significantly from that observed with CW recording. While CW enables faster polymerization and higher risk of overmodulation, pulsed regimes are more affected by dye absorption and oxygen inhibition. Consequently, reducing the dye concentration is necessary to minimize surface absorption and to improve in-depth grating uniformity. Moreover, lowering the pulse repetition frequency decreases the threshold energy required to initiate photopolymerization, resulting in a more efficient recording.

The analysis of the second-order angular response further reveals that nonlinearities also arise from the temporal structure of the exposure. Lower repetition rates tend to produce gratings with more linear refractive index profiles, whereas pulsed exposure generally results in more attenuated gratings compared to those recorded under CW conditions. Overall, these findings highlight the importance of optimizing the dye concentration, repetition rate, and exposure conditions to achieve efficient and uniform holographic gratings in PVA/AA photopolymers, particularly when employing pulsed laser sources.

Future work should focus on photopolymerization dynamics and on the development of models capable of accurately describing and validating the material response in this regime. These efforts will provide a deeper insight into the interplay between diffusion and polymerization processes during pulsed exposure, contributing to a more comprehensive understanding of the phenomena reported in this study.

## Figures and Tables

**Table 1:** Composition of PVA/AA solutions for different YE concentrations.

Components	Type 1	Type 2	Type 3
PVA	8% *w*/*w* H_2_O		
AA	0.404 M		
MBA	0.055 M		
TEA	0.309 M		
YE	$19.6\cdot 10^{-5}$ M	$9.88\cdot 10^{-5}$ M	$6.60\cdot 10^{-5}$ M

**Table 2:** Fitting parameters of photopolymer samples exposed under pulsed light for different concentrations of YE shown in Figure 2.

Parameters	Type 1	Type 2	Type 3
Δn	0.00250	0.00250	0.00248
α (${µm}^{-1}$)	0.0300	0.0190	0.0150
$d_{\mathrm{optic}}$ (µm)	34.2	36.7	47.8

**Table 3:** Fitting parameters of photopolymer Type 3 exposed at different repetition rates as shown in Figure 3 and reconstructed in Figure 4.

Parameters	CW	10 Hz	5 Hz	2.5 Hz	1 Hz
Δn	0.00255	0.00240	0.00235	0.00255	0.00250
α (${µm}^{-1}$)	0.0120	0.0150	0.0160	0.0175	0.0185
$d_{\mathrm{optic}}$ (µm)	49.9	43.6	44.5	40.9	38.6

## Data Availability

The original contributions presented in this study are included in the article. Further inquiries can be directed to the corresponding author.
